# Tumor suppressor NPRL2 induces ROS production and DNA damage response

**DOI:** 10.1038/s41598-017-15497-0

**Published:** 2017-11-10

**Authors:** Yinxing Ma, Licia Silveri, John LaCava, Svetlana Dokudovskaya

**Affiliations:** 1 CNRS UMR 8126, Université Paris-Sud 11, Institut Gustave Roussy, 114, rue Edouard Vaillant, 94805 Villejuif, France; 2 Laboratory of Cellular and Structural Biology, The Rockefeller University, New York, New York, USA; 3 Institute for Systems Genetics and Department of Biochemistry and Molecular Pharmacology, New York University School of Medicine, New York, New York, 10016 USA

## Abstract

The SEA/GATOR complex is an essential regulator of the mTORC1 pathway. In mammals the GATOR1 complex is composed of the proteins DEPDC5, NPRL2 and NPRL3. GATOR1 serves as an mTORC1 inhibitor and activates the mTORC1-modulating RagA GTPase. However, several GATOR members have mTORC1 independent functions. Here we characterize mammalian cells overexpressing the GATOR1 component NPRL2. We demonstrate that, in the cells with active p53, ectopic expression of NPRL2 induces NOX2-dependent production of reactive oxygen species and DNA damage. Overexpressed NPRL2 accumulates in the nucleus, together with apoptosis-inducing factor (AIF). These events are accompanied by phosphorylation of p53, activation of a DNA-damage response and cell cycle arrest in G1 phase, followed by apoptosis. In the cells negative for active p53, NPRL2 ectopic expression leads to activation of CHK1 or CHK2 kinases and cell cycle arrest in S or G2/M phases. Combined, these results demonstrate a new role for the NPRL2, distinct from its function in mTORC1 regulation.

## Introduction

The mammalian (or mechanistic) target of the rapamycin complex (mTORC1) is a key regulator of cellular homeostasis and survival upon different stresses^1,2^. The NPRL2 and NPRL3 proteins, as part of the GATOR1 complex, have recently been described as essential controllers of the mTORC1 pathway^3,4^. This paradigm was first established for their yeast orthologues, Npr2 and Npr3^5^. Nitrogen permease regulators 2 and 3 specifically responded to amino acid deprivation by inactivation of Tor1 kinase. Not surprisingly, deletion of Npr2 and Npr3 led to defects in autophagy^6–9^, a catabolic process tightly controlled by mTORC1. In yeast, Npr2 and Npr3 are the members of the SEA complex, containing also a number of yet poorly characterized proteins with structural elements shared with intracellular trafficking complexes^4,6,10^. Three proteins of the SEA complex – Sea1, Npr2, Npr3 – comprise the SEACIT subcomplex, which inhibits TORC1 signaling, while five others – Sea2, Sea3, Sea4, Seh1, Sec. 13 – are the members of SEACAT subcomplex, which activates TORC1 pathway^11,12^. The same mode of mTORC1 regulation was reported for the mammalian SEA orthologue - GATOR1/GATOR2^3^. The GATOR1 complex is composed of NPRL2, NPRL3 and the Sea1 homologue DEPDC5, and exhibits a GTPase activating (GAP) function on an essential mTORC1 modulator, the RagA GTPase. However, in contrast to yeast, where all eight components form a stable SEA complex^13^, GATOR1 and GATOR2 are weakly associated in mammalian cells^3^ and require additional mammalian-specific factors to maintain their communication^14,15^. GATOR1 members are the most intensively studied and the role of NPRL2 and NPRL3 in the regulation of the mTORC1 pathway has been reported for fission yeasts^16^, *C*.*elegans* ^17,18^, *Drosophila* ^19–22^ and mice^23^.

The molecular functions of NPRL2, NPRL3, and their orthologues remain under explored. Both proteins exhibit similar structures and may be paralogues. At their N-termini they contain a longin domain, which is often found in guanine nucleotide exchange factors (GEFs); however GEF activity has not yet been demonstrated for these proteins^24,25^. At their C-terminus NPRL2 and NPRL3 contain two or more winged helix-turn-helix (HTH) domains, which facilitate interactions with DNA. Notably, NPRL2 and NPRL3 are typically maintained at the very low levels within cells. For example, protein expression level of NPRL2 is between 100 and 10000 times lower than GAPDH (depending on a tissue), as annotated by Human Integrated Protein Expression Database (HIPED)^26^ (http://www.genecards.org). NPRL2 and NPRL3 overexpression inhibits cell proliferation^27–29^.

The members of GATOR1 have the properties of tumor suppressors^3^. A lot of data has been collected for NPRL2 (also called Tumor suppressor candidate 4 - TUSC4), which was suggested to be a tumor suppressor more than a decade ago^30^. Although NPRL2 is expressed in many normal tissues, inactivation of the *NPRL2* gene has been found in many human cancers and cancer-derived cell lines, supporting a linkage to oncogenesis^3,4,28,31^. Low expression of NPRL2 in different types of lung cancers was correlated with resistance to cisplatin, one of the primary chemotherapeutics for lung cancer^32^. Overexpression of NPRL2 overcomes cisplatin resistance in NPRL2 deficient cells through the activation of the DNA damage checkpoint pathway^29^. However, the consequences of NPRL2 overexpression to the cell fate have not been studied in details.

Here we characterize mammalian cells lines overexpressing NPRL2 and NPRL3. Proteomic analysis of HEK293 cells stably expressing FLAG-NPRL2-GFP reveals interactions with mitochondria and proteins involved in the DNA damage and repair. We further demonstrate that the overexpression of NPRL2 induces Nox2-dependent production of reactive oxygen species, inhibits cell viability, and induces cell cycle arrest and apoptosis. Overexpressed NPRL2 accumulates in the nucleus together with apoptosis-inducing factor (AIF). These events are accompanied by activation of p53 signaling pathway and DNA-damage response. Ectopic expression of NPRL2 in the cells negative for active p53 activates CHK1 or CHK2 kinases and results in the cell cycle arrest in S or G2/M phases.

## Results

### NPRL2 and NPRL3 are nucleo-cytoplasmic proteins

**Figure 1:**
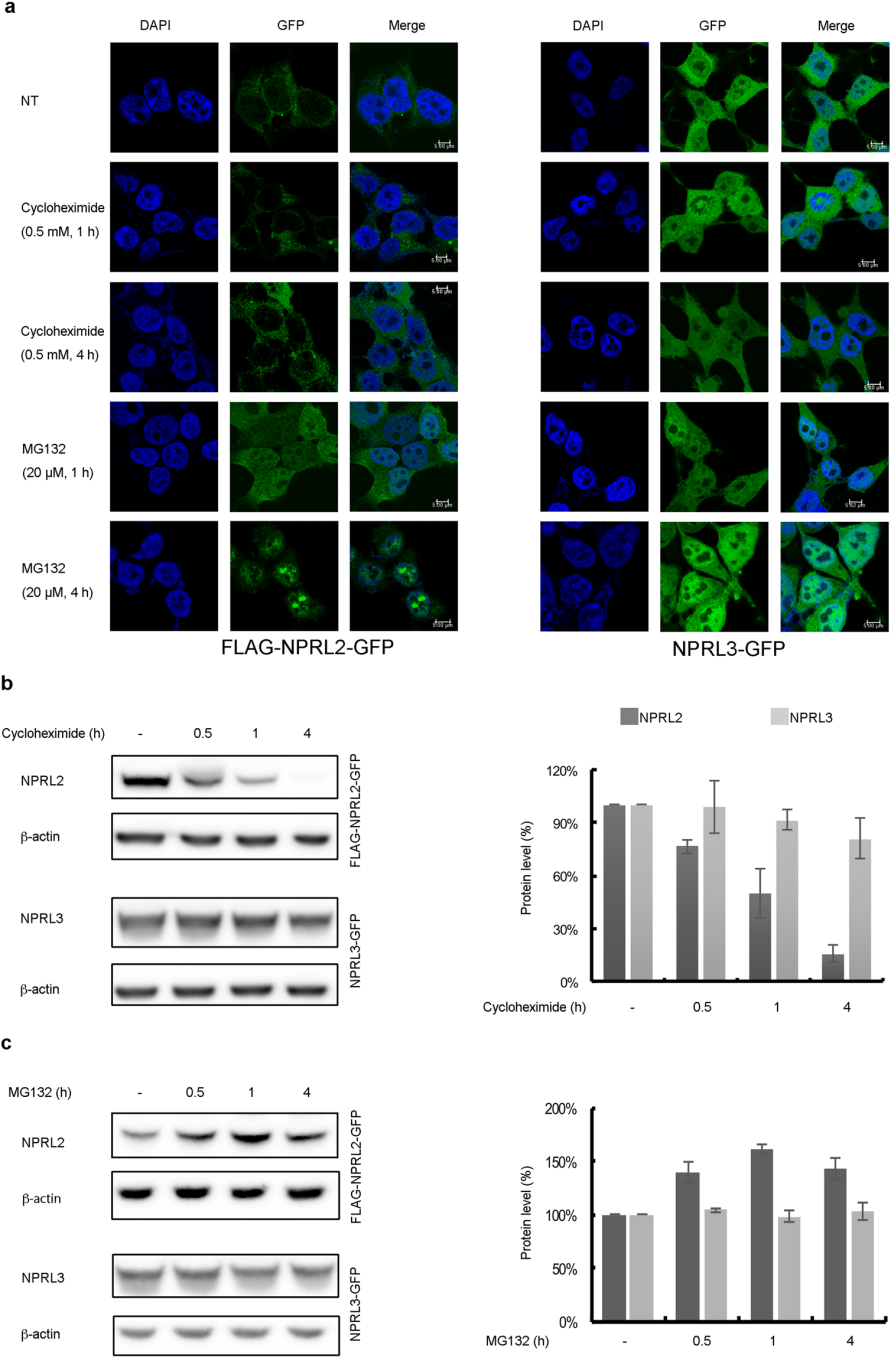
NPRL2 and NPRL3 are nucleo-cytoplasmic proteins. (**a**) GFP signal HEK293 cells stably expressing FLAG-NPRL2-GFP (left panel) or NPRL3-GFP (right panel) was observed in the cells without treatment (NT) or in the cells treated with cycloheximide or MG132 at indicated concentrations and time. Scale bar is 5 μm. (**b**) Treatment with cycloheximide reduces NPRL2 protein level, but does not change NPRL3 expression. Whole cell lysates from the cells stably expressing FLAG-NPRL2-GFP or NPRL3-GFP and treated with cycloheximide for indicated time were analyzed by Western blotting with anti-GFP antibody to detect appropriate GATOR1 component and with anti-β-actin as a loading control. The protein level in % was calculated by normalizing the GFP signal to a corresponding β-actin signal and represented as a graph. The signal in the samples without treatment was set at 100%. Error bars represent the standard deviation in three independent experiments. (**c**). Treatment with MG132 induces NPRL2 stability. The analysis of cells treated with 20 μM MG132 for indicated time was done as in (**b**).

To study the effects of NPRL2 and NPRL3 overexpression we established human embryonic kidney HEK293 cell lines stably expressing these proteins fused with FLAG or GFP from the EF1 promoter (accordingly, FLAG-NPRL2-GFP and NPRL3-GFP). In these cells, the amount of mRNAs coding for each protein were found to be ~200 times higher than in the wild type HEK293 cells (data not shown). We were not able to compare the levels of the NPRL2/3 proteins present in the wild type and modified cells because currently available commercial antibodies do not detect endogenous proteins by Western blotting^4,6,15^. A test of cell proliferation and viability (see Methods) did not reveal any differences between wild type and our HEK293 cells constitutively expressing NPRL2 or NPRL3 fusions (data not shown). Immunofluorescence experiments showed that FLAG-NPRL2-GFP was located mainly in the cytoplasm with some residual staining in the nucleus while NPRL3-GFP was rather equally distributed between the nucleus and the cytoplasm (Fig. 1a) in agreement with nucleo-cytoplasmic staining reported for overexpressed NPRL3 during its initial characterization^27^.

Low steady state levels of endogenous NPRL2 and NPRL3 may be explained by rapid turnover. To check this possibility, we treated cells stably expressing NPRL2 and NPRL3 fusions with the protein synthesis inhibitor cycloheximide (CHX). While the level of NPRL3 was not apparently greatly affected, the amount of NPRL2 was significantly decreased already by 30 min of treatment (Fig. 1b). After 4 h of treatment NPRL2 was completely degraded. Further analysis has indicated that NPRL2 half-life time was approximately 15 min (data not shown).

Cycloheximide treatment induces mTORC1 activation and inhibits autophagy during nitrogen starvation^33,34^. The canonical model suggests that CHX causes mTORC1 activation through the accumulation of intracellular amino acids, resulting from inhibition of the protein synthesis. However, this might not be the only explanation. Our data show that CHX treatment greatly reduces the amount of the mTORC1 inhibitor NPRL2, resulting in mTORC1 reactivation. Indeed, when we checked mTORC1 activity by monitoring the level of p70 phosphorylation, we observed that declining of the NPRL2 level caused by CHX treatment was correlated with the increase of mTORC1 activity (Supplementary Fig. S1a). Inversely, overexpression of NPRL2 reduces mTORC1 activity (Supplementary Fig. S1b).

We further verified whether NPRL2 degradation was mediated by proteasome, by treating the cells with a proteasome inhibitor, MG132 (Fig. 1c): the NPRL3 level did not change, while the amount of NPRL2 significantly increased, consistent with NPRL2 degradation by the proteasome. Moreover, MG132 treatment resulted in nuclear retention of NPRL2 (Fig. 1a). Thus our data demonstrate that NPRL2 and NPRL3 are nucleo-cytoplasmic proteins. The endogenous levels of both proteins are very low and in the case of NPRL2 it can be explained by rapid turnover of the protein via proteasome-mediated degradation.

### GATOR1 proteome reveals interactions with mitochondria and proteins involved in the DNA damage and repair

**Figure 2:**
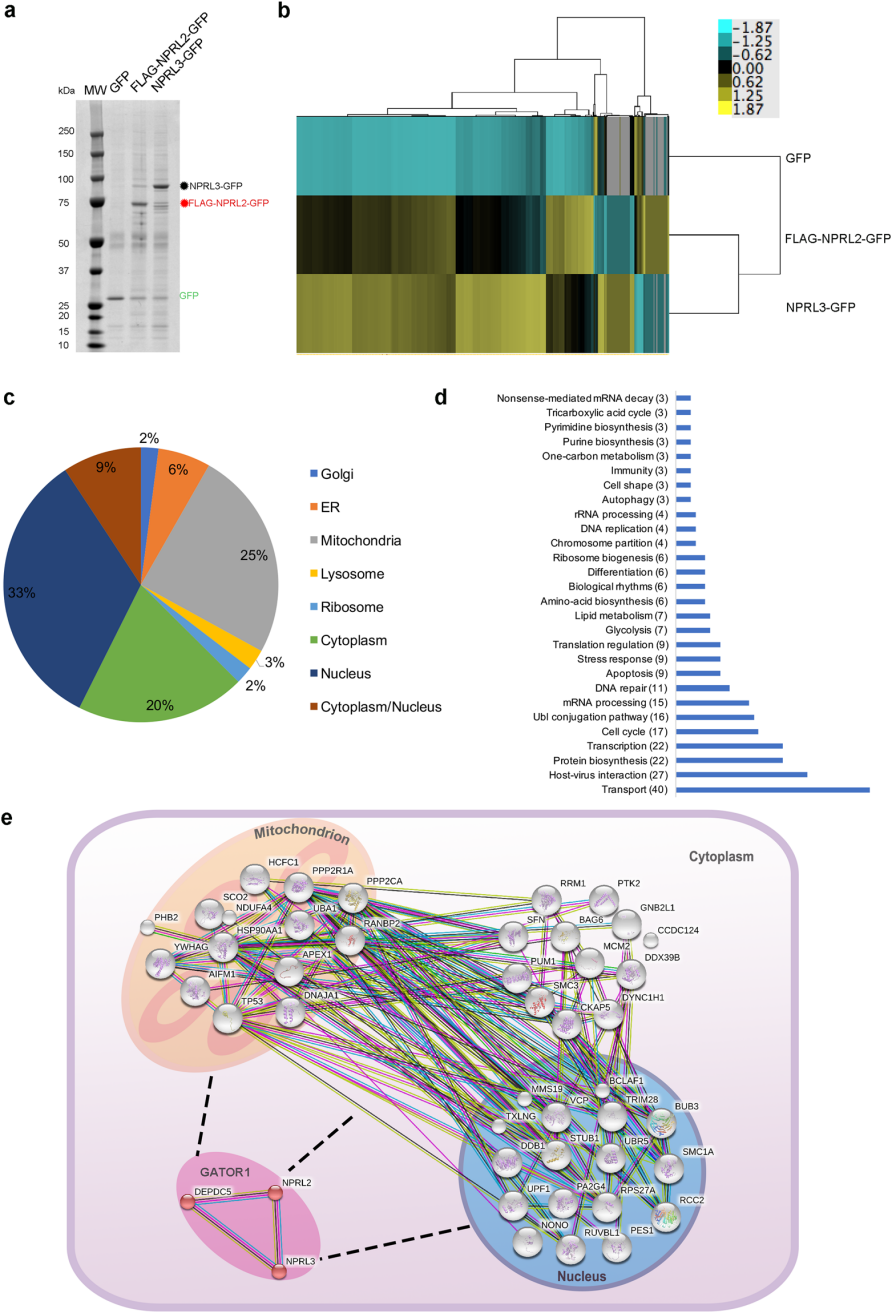
Analysis of NPRL2 proteome. (**a**) Affinity purification of GFP, FLAG-NPRL2-GFP and NPRL3–GFP from corresponding HEK293 cells stably expressing these proteins was performed as described in Methods. Co-precipitating proteins were resolved by SDS-PAGE and visualized by Coomassie blue staining. (**b**) Heat map comparing proteins co-precipitating with GFP, FLAG-NPRL2-GFP and NPRL3–GFP. Shown are color-coded relative protein abundances (see Methods). (**c**,**d**) Classification of proteins co-purified with NPRL2 according to their localization (**c**) and biological process (**d**) was done by Gene Ontology Database. (**d**) Protein interaction networks of GATOR1 complex. The interaction networks between the GATOR1 complex and selected proteins from NPRL2 interactome known to be involved in the processes of DNA damage response (Supplementary Table S3) was done using protein interaction information from the STRING database (https://string-db.org/).

To get information about NPRL2 and NPRL3 interactors beyond their immediate partners in the GATOR complex, we performed proteomic analysis of immunoprecipitates (IPs) obtained from HEK293 cells stably expressing FLAG-NPRL2-GFP or NPRL3-GFP. We first compared 24 different extraction solutions comprising different combinations of buffers, salts, and detergents at various concentrations (Supplementary Fig. 2a and b, Supplementary Table S1). We compared the protein profiles obtained by SDS-PAGE and found that a solution described in the initial purification of GATOR components^3^ (see Methods) provided a relatively protein-rich IP (Supplementary Fig. S2, lane 1). As judged by total protein staining, a comparably protein-rich affinity purified fraction was also obtained when using another solution (Supplementary Fig. S2, lane 17). However, upon comparison of these two affinity purified fractions by mass spectrometry, we observed that solution N17 yielded fewer peptides and lower confidence scores for members of the GATOR1 complex and several other known interactors (data not shown). We therefore used solution N1 to perform a large-scale purification from HEK293 cells stably expressing FLAG-NPRL2-GFP or NPRL3-GFP and GFP as a control (Fig. 2a).

Because NPRL2 and NPRL3 have similar set of interacting partners (Fig. 2b, Supplementary Table S2), we concentrated on the analysis of the NPRL2 proteome, containing 291 proteins. The majority of NPRL2 interactors resided in the cytoplasm, however 33% of the proteins had nuclear localization and 9% are described as nucleo-cytoplasmic proteins (Fig. 2c). As expected, the proteins most highly co-enriched with NPRL2 were the members of the GATOR1 complex – NPRL3 and DEPDC5 (Supplementary Tables S2 and S3). Components of GATOR2 complex were also present in the IP (Supplementary Table S3), but in smaller amounts. This is consistent with previous observations that GATOR1 and GATOR2 associate weakly with one another in mammalian cells, in contrast to their yeast counterparts from the SEA complex^3,13^. We did not detect any components of the mTORC1 complex and only very few lysosomal proteins. One of the explanations may be that the GATOR1 interaction with mTORC1 at the lysosome is enhanced by stress conditions^3^, which was not the case in this proteomic study. The main cytoplasmic interactors of NPRL2 were ribosomal and various other proteins implicated in different metabolic processes including amino acid biogenesis, glycolysis, lipid metabolism, etc. (Fig. 2d). The majority of the nuclear proteins interacting with NPRL2 were those involved in DNA damage and repair, transcription, and RNA processing (Fig. 2d). Analysis of biological pathways revealed that the majority of the proteins participate in signal transduction and metabolic pathways, including cellular responses to stresses, and control of DNA replication, gene expression, and cell cycle (Supplementary Fig. S2,c). Overall the strongest interactors of NPRL2 (Supplementary Table S3) constitute a highly interconnected network (Fig. 2e), formed by cytoplasmic, mitochondrial and nuclear proteins.

### Overexpression of NPRL2 promotes interaction with AIF, induces apoptosis and DNA damage

15% of the proteins (43 out of 291) detected in both NPRL2 and NPRL3 pullouts had functions related to DNA damage (Fig. 2d and e, Supplementary Table S3), including proteins residing in the mitochondrion or in the nucleus (Fig. 2e). Moreover, both NPRL2 and NPRL3 interact with apoptosis-inducing factor (AIF), which is involved not only in the maintenance of mitochondrial morphology, energy metabolism and apoptosis, but also known to have important role during DNA damage^35^. In the case of NPRL2 the number of AIF peptides detected in the IP was comparable to the number of peptides detected for bona-fide NPRL2 partners NPRL3 and DEPDC5 (Supplementary Tables S2 and S3). We therefore decided to further study the NPRL2/AIF interaction.

**Figure 3:**
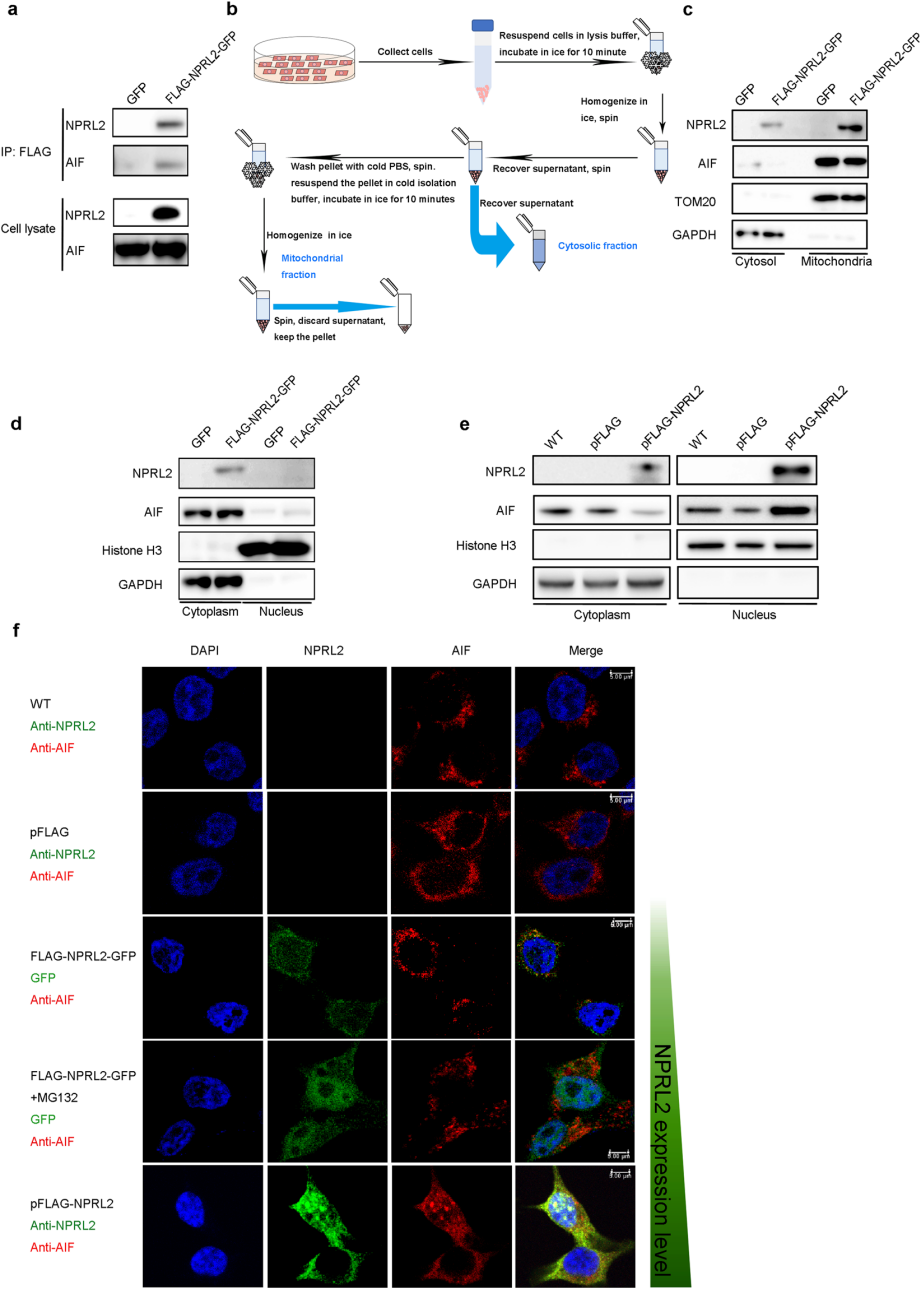
Overexpression of NPRL2 promotes interaction with AIF and induces AIF translocation to the nucleus. (**a**) NPRL2 interacts with AIF. Immunoprecipitates were prepared from HEK293 cells stably expressing GFP or FLAG-NPRL2-GFP and analyzed by immunoblotting for the indicated proteins. (**b**) The scheme of subcellular fractionation experiment used for the detection of NPRL2 localization. (**c**) Overexpressed NPRL2 localizes to mitochondria. The subcellular localization of NPRL2 was detected by immunoblotting with antibodies against indicated proteins in the mitochondrial and cytosolic extracts. TOM20 was used as mitochondrial marker and GAPDH as cytosolic marker. (**d**–**f**) Overexpression of the NPRL2 induces re-localization of NPRL2 and AIF to the nucleus. The cytoplasm and nuclear extracts were prepared from HEK293 cells either stably expressing FLAG-NPRL2-GFP or GFP (**d**) or transfected with pFLAG or pFLAG-NPRL2 plasmids (**e**) and analyzed by immunoblotting for the indicated proteins with Histone H3 as a marker for the nucleus and GAPDH as a marker for the cytoplasm. (**f**) Immunofluorescence analysis of localization of NPRL2 and AIF localization. Nucleus is stained with DAPI (blue); NPRL2 (green) is detected either via GFP in case of HEK293 stably expressing FLAG-NPRL2-GFP or with immunofluorescence with anti-NPRL2 antibody; AIF (red) is detected with anti-AIF antibody. Scale bar, 5 μm. The increased level of NPRL2 expression in these experiments is indicated with growing green bar to the right of image panels.

To confirm the results obtained by IP and mass spectrometry, we performed western blotting, which revealed that cells stably expressing FLAG-NPRL2-GFP co-precipitated AIF, in contrast to the cells expressing GFP-only, which did not (Fig. 3a). AIF can be found in multiple locations in the cells^35^: in healthy cells, AIF resides in the mitochondrial intermembrane space, where it acts as an NADH-dependent oxidoreductase. If the cell enters apoptosis, AIF undergoes N-terminal proteolysis and is released first to the cytoplasm, followed by re-location to the nucleus where it induces chromatin condensation and DNA degradation. Subcellular fractionation experiments (Fig. 3b) revealed that in the cells stably overexpressing FLAG-NPRL2-GFP, a portion of cytoplasmic pool of this protein is localized to the mitochondria, where it interacts with AIF (Fig. 3c). In these cells, the major pool of NPRL2 and AIF localized in the cytoplasm and not in the nucleus (Fig. 3d). Treatment with MG132, which increases the cellular level of NPRL2 at about 1.5 times (Fig. 1b), is sufficient to relocate NPRL2 to the nucleus (Figs 1a and 3f), but not enough to induce AIF nuclear localization (Fig. 3f). However, in cells expressing NPRL2 from a plasmid with a strong CMV promoter, AIF relocates to the nucleus where it co-localizes with NPRL2 (Fig. 3e and f). Overexpression of NPRL2 at high levels promotes nuclear localization of NPRL2 (Figs 1a and 3f), yet the mTORC1 activity in such cells is also reduced (Supplementary Fig. S1), in agreement with recently published studies^15^. At the same time, elevated amounts of NPRL2 do not have an effect on the expression of fellow GATOR1 component NPRL3 (Supplementary Fig. S1B).

**Figure 4:**
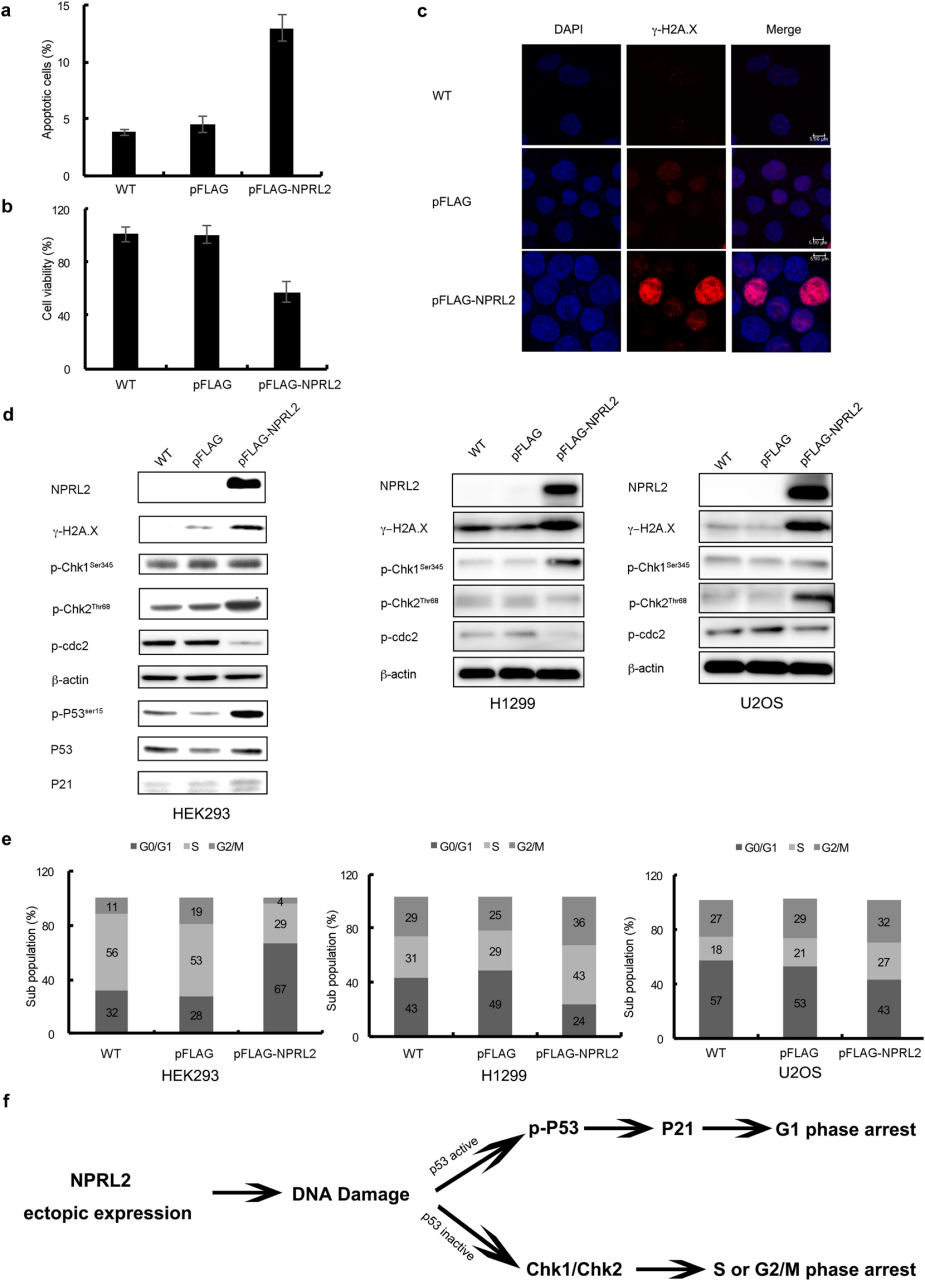
Overexpression of NPRL2 induces apoptosis, DNA damage and DNA damage response. The percentage of apoptotic cells (**a**) and the analysis of cell viability (**b**) was calculated for wild type HEK293 cells (WT) and for cells transfected with 1 μg of pFLAG or pFLAG-NPRL2 plasmids for 24 h (see Methods for details). Error bars indicate standard deviations of the mean in three independent experiments (p-value < 0.01). (**c**) Immunofluorescence analysis of γ-H2A.X in wild type HEK293 cells (WT) and in cells transformed with 1 μg of a plasmid expressing pFLAG or pFLAG-NPRL2 for 24 h. (**d**) Whole cell extracts prepared from HEK293, H1299 and U2OS wild type cell lines (WT) or cell lines transfected with 1 μg of pFLAG or pFLAG-NPRL2 plasmids for 24 h were tested by Western blot with indicated antibodies. (**e**) The distribution of sub-populations of cells from **(d)** in different stages of cell cycle. (**f**) The schematic representation of the DNA damage response induced by NPRL2 overexpression, depending on the presence or absence of p53.

It is known that AIF nuclear localization is a signature of the apoptotic cells^35^. Therefore, we verified the level of apoptosis in the cells over-expressing NPRL2 from the strong CMV promoter. We found that the amount of apoptotic cells increased almost 3 times (Fig. 4a) and cell viability decreased by a factor of two (Fig. 4b).

AIF in the nucleus is proposed to directly interact with DNA and disrupt chromatin structure, thus participating in the DNA damage and preventing DNA repair. The hallmark of DNA damage is the strong increase of H2AX histone phosphorylation at Ser139, resulting in appearance of so-called γ-H2AX protein^36^. It is well established that DNA damage response (DDR) activates tumor suppressor protein p53 via phosphorylation at Ser15 and Ser20^37^. Activated p53 will induce expression of cyclin-dependent kinase inhibitor p21, which will block cyclin-dependent kinase 2 (CDK2), followed by cell cycle arrest in G1 phase. p21 can also inhibit cyclin-dependent kinase1 (CDC2), which leads to the cell cycle arrest in G2-M phase^38^. DDR is also regulated via ataxia telangiectasia mutated (ATM) and ataxia telangiectasia and Rad3 related (ATR) proteins, which can also phosphorylate and activate checkpoint kinases Chk1 and Chk2 respectively^39^.

In order to analyze DNA damage caused by NPRL2 ectopic expression, we used three cell lines with different status of p53: 1) HEK293 cells expressing constitutively active p53; 2) H1299 non-small lung cancer cells, in which p53 is deleted^40^ and 3) U2OS osteosarcoma cells, where p53 is inhibited by MDM2. MDM2 binding to p53 inhibits its transcription factor and tumor suppressor properties^41,42^ and results in the p53 proteolytic degradation by the ubiquitin-proteasome system^43^. Importantly, MDM2 is overexpressed in the U2OS cell line, in contrast to HEK293^44^. Therefore, even though some portion of p53 may be degraded by MDM2 in HEK293 cells, a fraction of active p53 remains.

Our data show that ectopic expression of NPRL2 results in DNA damage in all three cell lines tested. However, the DNA damage response mechanisms are different (Fig. 4d–f). When p53 is active (HEK293 cells), the cellular response to the NPRL2 overexpression is likely to be orchestrated via the p53 pathway: phosphorylated p53 causing p21 activation and cell cycle arrest in G1. CDC2 is inhibited in these cells in a dosage-dependent manner (Supplementary Fig. S3). CHK1 phosphorylation was not observed in HEK293 cells, and CHK2 phosphorylation was only slightly induced. In p53-negative H1299 cells, CHK1 phosphorylation was strongly induced and cells arrested in S phase (50% more in comparison to non-treated cells), in agreement with previous results^29^. Conversely, the CHK1 phosphorylation is nearly unaffected in U2OS cells, while CHK2 phosphorylation is elevated, resulting in 30% more S phase arrests compared to non-treated cells. Thus DDR response in the cells overexpressing NPRL2 is likely to be mediated via p53, while in p53 negative cells DDR is taken over by ATM/ATR kinases pathway (Fig. 4f).

### Overexpression of NPRL2 promotes ROS production

**Figure 5:**
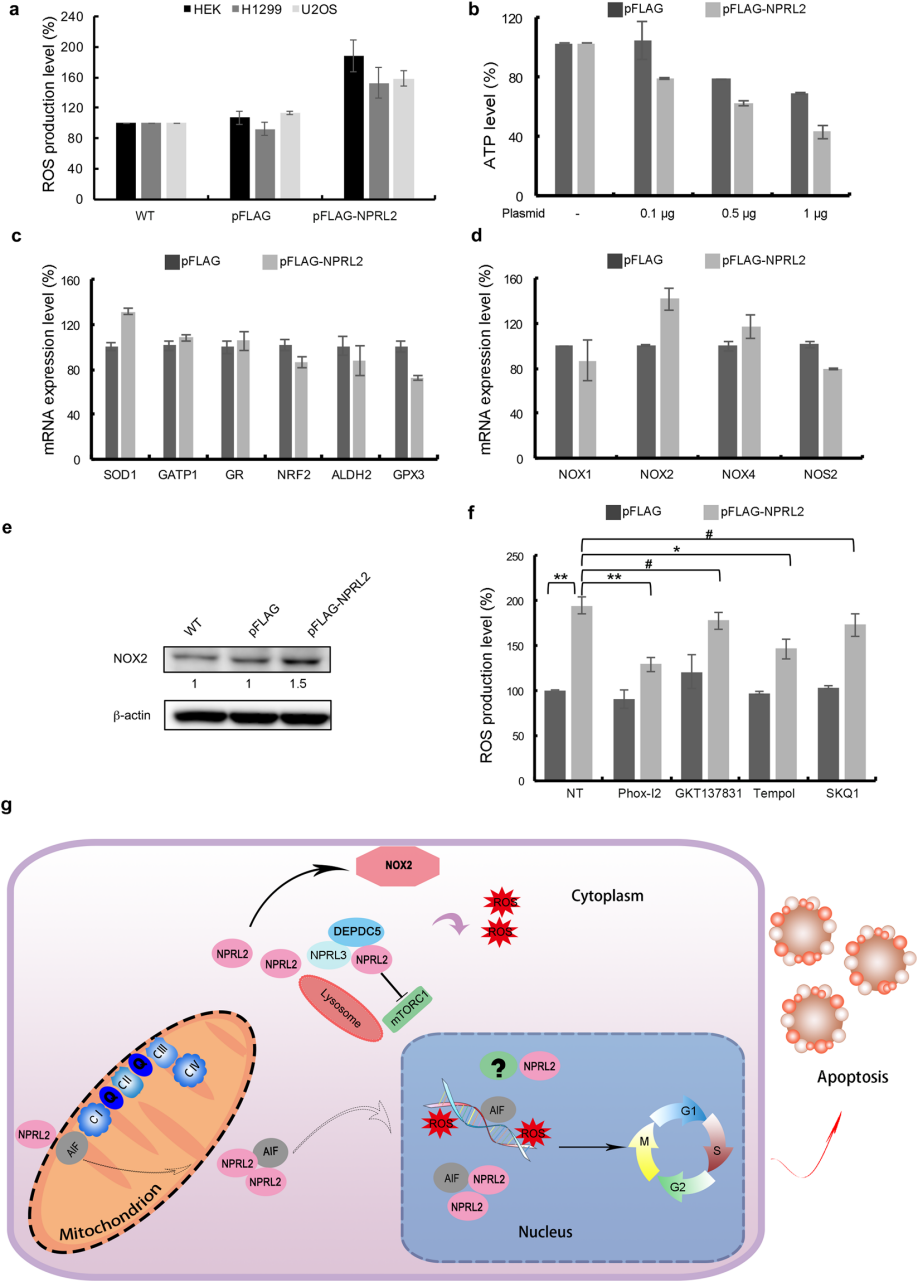
NPRL2 overexpression induces oxidative stress. (**a**) ROS production was estimated by measuring DHE level in non-treated HEK293, H1299 or U2OS cells (NT), and in cell transformed with 1 μg pFLAG or pFLAG-NPRL2 plasmids for 24 h. (**b**) Analysis of ATP production in HEK293 cells expressing pFLAG and pFLAG-NPRL2 plasmids at indicated concentrations during 24 h. (**c**) Analysis of mRNA expression levels of indicated genes in the HEK293 cells transformed with 1 μg of plasmids expressing pFLAG or pFLAG-NPRL2 during 24 h. (**d**) Analysis of mRNA expression levels of *NOX1*, *NOX2*, *NOX4* and *NOS2* in the HEK293 cells transformed as in (**c**). **(e)** Analysis of NOX2 expression level in the HEK293 cells transformed as in (**c**). An increase of NOX2 expression detected by Western blot is marked by a number at the bottom of an appropriate blot. (**f**) Analysis of ROS production (as in (**a**)) in the HEK293 cells treated with different antioxidants during 3 hours prior to pFLAG or pFLAG-NPRL2 overexpression. **p-value < 0.001; *p-value < 0.05; ^#^p-value > 0.05. For experiments in (**a**–**d**,**f**) error bars indicate standard deviations of the mean in three independent experiments (see also Supplementary Table S4 for all the statistics corresponding to the graphs in **a**–**d** and **f**). (**g**) Schematic representation of the effects of the NPRL2 overexpression. NPRL2 overexpression enhances mTORC1 suppression and induces ROS production via NOX2 activation. NPRL2 interacts with AIF and translocates to the nucleus, where AIF is loaded to the sites of DNA damage initiated by ROS. NPRL2 in the nucleus might also interact with other yet unknown factors (marked by “?”). DNA damage leads to the cell cycle arrest and eventual apoptosis.

One mechanism of DNA damage is increased production of the reactive oxygen species (ROS). We thus checked whether NPRL2 ectopic expression could induce ROS production. Indeed, cells overexpressing NPRL2 from CMV promoter exhibited very high level of ROS production in all three cell lines tested (Fig. 5a), comparable with the level achieved during treatment with hydrogen peroxide (Supplementary Fig. S3). ROS in the cells can be generated through various mechanisms. The principal sources of ROS are 1) leakage of electrons from mitochondrial respiratory chain because of mitochondria damage; 2) deficiency of the antioxidant system; or 3) increased activity of ROS-producing enzymes, such as NADPH oxidases (NOXs) and nitric oxide synthases NOSs^45^. We have tested whether NPRL2 overexpression can trigger activation of each of these pathways.

First, we checked the level of ATP and found that it is greatly reduced during NPRL2 overexpression in a dosage-dependent manner, indicating an elevated level of damaged mitochondria (Fig. 5b). In addition, NPRL2 overexpression triggers AIF release (see above), which is a sign of increased mitochondrial membrane permeability^46^.

Second, we verified the mRNA level different components of the antioxidant system and found that four of them (*NRF2*, *GSTP1*, *GR*, and *ALDH2*) did not change significantly, suggesting that the anti-oxidant system controlled by these factors probably functions normally (Fig. 5c). However, we detected an increased mRNA expression of superoxide dismutase (SOD1), localized at the outer mitochondrial membrane and involved in the destruction of superoxide radicals. Because intracellular superoxide is generated through the oxidation of NADPH by NOXs, it prompted us to examine the status of various NOXs.

Third, we checked the expression of several members of NOX family and detected that NOX2 was increased both at the mRNA and protein level (Fig. 5d and e). In addition, we detected a slight decrease in the level of glutathione peroxidase 3 (GPX3), an antioxidant enzyme, which catabolizes hydrogen peroxide. This supports an inverse relationship between NOX2 and GPX3, as reported recently^47^. We then verified the expression level of NOS2, which is involved in the generation of different reactive oxygen species – nitric oxide. The level of expression of NOS2 was not changed significantly, indicating that NPRL2 is manly activating a brunch of ROS, related to the superoxide production. Finally, we treated cells with different anti-oxidants, such as tempol, a general purpose redox cycling agent, SKQ1 – a potent mitochondria targeted antioxidant, GKT137831 - a dual NOX1/NOX4 inhibitor^48^, and Phox-I2 – a specific NOX2 inhibitor^49^ (Fig. 5f). Our results show that the strongest ROS inhibition in pFLAG-NPRL2 transfected HEK293 cells was observed during Phox-I2 treatment, indicating that NPRL2 overexpression mainly affects ROS production via NOX2 activation.

In summary, we showed that NPRL2 overexpression induces ROS production via impaired mitochondrial function and NOX2 activation. This leads to DNA damage, cell cycle arrest and apoptosis.

## Discussion

In the healthy cells expression of GATOR1 component NPRL2 is maintained at a very low level. Inactivation of NPRL2 leads to the impaired mTORC1 signaling^3^, very often observed in cancer cells and associated with anti-cancer drug resistance. Overexpression of NPRL2 overcomes drug resistance, but is toxic for the cells^29,32^. In this study we determined a mechanism for such cytotoxicity.

Our findings show that strong overexpression of NPRL2 induces DNA damage. In the cells with constitutively active p53 an excessive amount of NPRL2 results in cell cycle arrest in G1 phase and eventual apoptosis (Fig. 4). The increased susceptibility of these cells to apoptosis might be facilitated through the interaction of NPRL2 with AIF (Fig. 3). Our data are in agreement with recent results concerning NPRL2 overexpression in colon cancer cells lines HT29 and HCT116, which leads to cell cycle arrest in G1 phase and apoptosis^50^. HT29 contains a p53 missense mutation Arg273His, but p53 remains somewhat active^51^, while HCT116 has wild type p53^52^. At the same time our data demonstrate that in the cells negative for p53 (deleted or suppressed), the DNA damage response triggered by NPRL2 ectopic expression leads to the activation of Chk1 or Chk2 kinases and cell cycle arrest in S or G2/M phases (see also^29^).

We further demonstrate that NPRL2 overexpression in p53-active cells leads to mTORC1 inhibition (Supplementary Fig. S1b) and high level of ROS via NOX2 activation and increased mitochondrial dysfunction (Fig. 5g). Reactive oxygen species have been found to either activate or inhibit mTORC1^53^. Low doses of ROS generally induce TORC1 activity, while high ROS concentrations inhibit TORC1. The mechanisms responsible for mTORC1 regulation by oxidative stress are cell-type and stimulus dependent. For example, mTORC1 activity can be modulated by ROS generated via mitochondrial depolarization, which involves cysteine oxidation^54^. Furthermore, under oxidative stress mTORC1 activation is mediated via downregulation of the TSC1-TSC2 complex^55^. Oxidative stress can also stimulate mTORC1 recruitment to the stress granules, which contributes to its reduced activity^56^. Our findings about NPRL2-induced NOX2-mediated ROS accumulation provide additional elements to the complex mechanisms regulating cellular responses to stresses.

So far the majority of the functional studies concerning SEA/GATOR components were carried out in the cells with deletions of SEA/GATOR proteins, which allowed establishing an important role of this complex in mTORC1 pathway regulation^3,4,6^. At the same time NPRL2 overexpression is toxic for the cell and the mechanisms leading to this cytotoxicity could reveal additional functions of the NPRL2 and its partners. Moreover, studying overexpression of GATOR members is also clinically relevant. First, since NPRL2 overexpression inhibits cell proliferation, it might be of potential interest to explore a possibility to control NPRL2 expression in anti-cancer therapy. Second, NPRL2 deletion is correlated to resistance to mainstream anticancer drugs cisplatin and doxorubicin^32,57^. Overexpression of NPRL2 in NPRL2-deficient and cisplatin-resistant lung cancer cells allowed to overcome drug resistance^32^, but not too much explanation to this phenomenon has been provided so far^29^. Third, although NPRL2 mRNA seems to be expressed at the same level in practically every tissue, the protein overexpression was detected in placenta, B-lymphocytes, frontal cortex, testis and ovary^26^ (http://www.genecards.org). Interestingly, mutations and deletions of NPRL2 and other GATOR1 members were detected in ovarian and brain cancers, as well as in specific forms of epilepsies^4^.

The results present here demonstrate a new role of the NPRL2, apart from its function in mTORC1 regulation. Interestingly, several SEA/GATOR components have been found to possess additional functions, beyond their activities in the mTORC1 pathway^4^. NPRL3, an NPRL2 orthologue, can be localized to PML nuclear bodies^27^. NPRL3 in the nucleus can interact with all members of the p53 family, in particular with p73. Similar to NPRL2, overexpression of NPRL3 inhibits cell proliferation. Moreover, GATOR2 component also have double lives. In *Drosophila* and HeLa cells, Wdr24 promotes lysosome acidification and autophagic flux^22^. In *Drosophila*, Mios can be localized in the nucleus and, together with Seh1, is required for the maintenance of the meiotic cycle and oocyte identity^58,59^. Finally, SEH1 and SEC13 are the members of the nuclear pore complex, where they belong to the same Nup84/Nup107 subcomplex. In addition, SEC13 is a member of the COPII coated vesicles. This may reflect the common evolutionary origin of nuclear pore complexes, vesicle coating complexes, tethering complexes, and the SEA/GATOR complex in a progenitor membrane-associated coating complex^60^.

Thus the SEA/GATOR complex is emerging as a multifunctional regulator of several cellular processes. Depending on the metabolic condition and its concentration the SEA/GATOR can either inhibit or promote cellular proliferation, thus playing an essential role in the cellular fate.

## Methods

### Materials

Reagents were obtained from the following sources: Dulbecco’s Modified Eagle Medium, DMEM (41966-029), Fetal Bovine Serum (10270-106) and Trypsin-EDTA, Phenol red (25300-054), Dynabeads M-270 Epoxy (143.02D), NuPAGE™ 10% Bis-Tris Protein Gels (NP0302BOX), MOPS SDS Running Buffer 20X (NP0001), LDS sample buffer (NP0007), TRIzol® Reagent (15596-026), SuperSignal™ West Pico Chemiluminescent Substrate (34080), RevertAid™ H Minus Reverse Transcriptase (EP0451), NE-PER™ Nuclear and Cytoplasmic Extraction Reagents (78833) were from Life Technologies/Thermo Fisher Scientific. Cycloheximide (1391) and MG132 (EU0033-A), DAPI (1050-A) were from Euromedex. Protease inhibitor cocktail (04693159001), Phosphatase inhibitor cocktail (04 906 837 001), Fast start SYBR Green Master (04673484001) and XTT assay kit (11465015001) were from Roche. JetPEI DNA transfection reagent (101-10N) was from Polyplus-transfection/Ozyme; Membrane Immobilon-P, PVDF (IPVH00010) was from Merck/Millipore; Annexin V Apoptosis Detection Kit eFluor® 450 (88-8006) was from eBioscience; Mitochondrial ToxGlo™ Assay kit (G8000) was from Promega; Hydroethidine [Dihydroethidium, DHE] (15200) was from AAT Bioquest; Fluoroshield Mounting Medium with DAPI (ab104139) was from Abcam; Phox-12 (SML-869) was from Sigma; Tempol (ALX-430-081) was from Enzo Life Sciences; GKT137831 (17764) was from Cayman Chemicals. SkQ1 is a kind gift from B. Chernyak (Moscow State University).

The antibodies were obtained from the following sources: antibodies against FLAG (F3165), β-actin (A1978) and NPRL2 (SAB2501073) were from Sigma; the antibody against NPRL3 (H0008131-A01) was from Abnova; the antibody against GFP (11814460001) was from Roche; antibodies against P70 pT389 (9206), P70 (9202), P53 pS15 (9284), P21 (2946); CDC2 pT15 (9111), GAPDH (2118), Histone H3 (4499), AIF (5318), TOM20 (42406), γ-H2A.X pS139/T142 (5438), Chk1 Ps345 (2341), Chk2 pT68 (2197) were from Cell Signaling Technology. The antibody against gp91-phox (C-15) (sc-5827) was from Santa Cruz Biotechnology; HRP- labeled anti-mouse (315035003) and anti-rabbit (111035144) secondary antibodies were from Jackson ImmunoResearch. Goat anti-Mouse IgG/IgA/IgM (H + L) Secondary Antibody, Alexa Fluor® 488 conjugate (A1-667) and Goat anti-Rabbit IgG (H + L) Cross-Adsorbed Secondary Antibody, Alexa Fluor 532 (A11009) were from Life Technologies/Thermo Fisher Scientific.

The plasmid pc5FLAG-wt-humanTUSC4 (pFLAG-NPRL2) and empty vector pc5FLAG was a generous gift from Dr. Fujita, (Cancer Chemotherapy Center of JFCK, Tokyo, Japan). A plasmid pcDNA3-HA-C16orf35 coding for NPRL3 isoform 1 was a generous gift from Dr. Collavin (LNCIB, Trieste, Italy).

### Cell lines and tissue culture

HEK293 were cultured in DMEM supplemented with 2 mM glutamine and 10% Fetal Bovine Serum and regularly tested for the presence of mycoplasma. Cell lines were maintained at humidified incubator at 37 °C and 5% CO_2_.

### Generation of cell lines stably expressing FLAG-NPRL2-GFP and NPRL3-GFP

Lentiviral vectors rLV-EF1-GFP, rLV-EF1-FLAG-NPRL2-GFP and rLV-EF1-NPRL3-GFP and corresponding lentiviruses were produced by Vectalys (www.vectalys.com). Plasmids pc5FLAG-wt-human-TUSC4 and pcDNA3-HA-C16orf35 (see above) were used to amplify cDNAs of NPRL2 and NPRL3. To obtain cells stably expressing GFP, HEK293 were plated in DMEM supplemented with 10% IFS one day prior to infection in order to reach 30% confluence at the day of the infection. The medium was changed and the cells were infected with appropriate lentiviruses at concentration of 40 lenviral particles per cell. 2–3 days after infection, cells were washed 3 times with PBS and single-cell sorted with a flow cytometer into DMEM, supplemented with 10% IFS and cultured for another 4 days. The protein expression was verified by western blot and fluorescence.

### Immunoprecipitation

The screen for optimal IP conditions was carried out as previously described^61^. Briefly, cells were cryo-milled, and the resulting frozen powder was distributed into a multi-well plate. 24 different extraction buffers (Supplementary Table 1) were rapidly added in parallel in wells containing ~50 mg of cell powder and the powder was resuspended completely by mechanical agitation and brief sonication. Insoluble materials were removed by centrifugation. Magnetic bead–based affinity capture experiment was performed on the clarified extract: each mixture was collected, washed with the appropriate extraction buffer, and eluted with LDS sample buffer after incubation at 75 °C for 10 min. The elutions were collected and subjected to SDS-PAGE followed by Coomassie blue staining to observe the protein co-precipitation profiles.

The large scale immunoprecipitation with selected extraction buffer (40 mM HEPES, pH 7.4, 2.5 mM MgCl_2_, 1% Triton X-100) was performed exactly as described in ref.^62^. Three technical replicates were performed for each immunoprecipitation. For one immunoprecipitation ~200 mg of appropriate HEK293 cells were used; clarified extracts were subjected to IP using in-house prepared polyclonal anti-GFP antibodies were coupled to Dynabeads as described in refs^63,64^. The elution from the IP was loaded on 4–12% Bis-Tris gels and run only 4–6 mm into the gel to produce a “gel plug”^65^. This small area of gel, containing all proteins present in the immunoprecipitate, was excised and subjected to in-gel with trypsin digest essentially as described earlier^64^. Briefly, gel plugs were cut into ~1 mm cubes for processing. Samples were destained, digested with trypsin, and desalted using reversed-phase OMIX tips (Agilent P/N A57003100), essentially as described in^64^ except that 50 µl 0.1% (w/v) trifluoroacetic acid was added to each tube to extract peptides from the gel pieces and the peptides were eluted from the tips first with 100 µl of 40% (v/v) ACN, 0.5% (v/v) acetic acid (E1) and then with 100 µl of 80% (v/v) ACN, 0.5% (v/v) acetic acid (E2). E1 and E2 were combined, frozen in liquid nitrogen, and dried down in a centrifugal vacuum concentrator.

### Mass spectrometry

Dried peptides were resuspended in 20 μl 5% v/v methanol, 0.2% v/v formic acid, and 5 μl were loaded to an EASY-Spray column (Thermo Fisher Scientific, ES800, PepMap C18, 3 μm bead diameter, 100 A pore, 75 μm column inner diameter × 15 cm length). Peptides were eluted with a linear gradient of 4–30% Solvent B in 55 minutes (Solvent A: 0.1% v/v formic acid in water, Solvent B: 0.1% v/v formic acid in acetonitrile) for online ESI-MS and MS/MS analyses with a Q Exactive Plus mass spectrometer (Thermo Fisher Scientific). MS/MS analyses of the top 10 precursors in each full scan used the following parameters: resolution: 17,500 (at 200 Th); AGC target: 1 × 10^5^; maximum injection time: 100 ms; isolation width: 2.0 m/z; normalized collision energy: 30%; charge >1; peptide match: preferred; dynamic exclusion length: 20 seconds. The raw files were processed with MaxQuant (version 1.5.8.3) and searched against the Uniprot Human protein database (158,090 entries in total). The following parameters were used for database searches: cysteine carbamido methylation was selected as a fixed modification; methionine oxidation, protein N-terminal acetylation, and phosphorylation on serine, threonine and tyrosine were selected as variable modifications. Up to two missing cleavage points were allowed. The precursor ion mass tolerances were 7 ppm, and fragment ion mass tolerance was 0.5 Da for MS/MS spectra. The false discovery rate (FDR) was set to < 1.0% for both peptide and protein identifications, the minimum peptide length was set to 6. The value for the label-free quantification (LFQ) intensity minimum ratio was set to 2. To retain high confidence interacting partners of NPRL2 and NPRL3 we excluded proteins, which were identified only in one replica and proteins, which were detected with 1–2 peptides. For the generation of the heat map by Perseus (version 1.5.8.5) (http://www.perseus-framework.org/), LFQ intensities were log_2_ transformed and z-score normalized before hierarchical clustering (Fig. 2b). Proteins were analyzed by Gene Ontology Database to get the information about cellular localization and function of GATOR1 interactors.

### Transfection

200,000 cells/well were plated in 12-well plate in complete DMEM medium. One day later when cells reached 50–70% confluence they were transfected with appropriate plasmid using the jetPEI reagent following manufacturer’s protocol. Transfection was carried out for 24 h.

### Subcellular fractionation

Cells were washed once in ice cold PBS (pH 7.4) the pellet was resuspended in the buffer (1 mM HEPES pH 7.4, 1 mM EDTA), incubated 10 min in ice, homogenized for 2 minutes on ice using Dounce homogenizer and centrifuged at 700 g for 10 min. The supernatant was recovered and centrifuged at 10.000 g for 30 min to obtain the cytosolic fraction. In order to obtain mitochondrial fraction the pellet was further washed with ice cold PBS and centrifuged 5 min at 450 g. After centrifugation, the pellet was resuspended in 1 mL of cold isolation buffer (75 mM sucrose, 20 mM HEPES, 225 mM mannitol, 0.5 mM EDTA, pH 7.2), placed on ice and homogenized for 2 minutes on ice with Dounce homogenizer followed by centrifugation at 750 g for 20 min. The pellet was discarded and the supernatant was centrifuged at 10.000 g for 10 min to obtain mitochondrial fraction. For separation of nucleus from cytoplasm the NE-PER™ Nuclear and Cytoplasmic Extraction Reagents were used according to the manufacturer’s instructions. The purity of the fractions was analyzed by immunoblotting with antibodies detecting bona-fide components of an appropriate cellular fraction.

### Whole cell lysates

The culture medium was removed and the cells washed once in cold PBS. Cold NETN buffer (150 mM NaCl, 1 mM EDTA, 50 mM Tris, pH 7.5, 0.5% NP-40, protease and phosphatase inhibitors) was added to a pellet and incubated on ice for 30 min. 100 μl of NTEN buffer for the well was added for 24-wells plate, 200 μl - for 12 wells-plate. The cellular suspension was sonicated, spun at 10.000 g, 10 min, 4 °C. The LDS sample buffer was added to a supernatant, incubated at 75 °C for 10 min and stored at −20 °C.

### RNA Isolation and gene expression analysis by quantitative real-time PCR

Total RNA were extracted using TRIzol® Reagent, according to manufacturer procedures. 1 μg of total RNA was used for reverse transcription in a 20 μl final reaction volume using the RevertAid™ H Minus Reverse Transcriptase following the manufacturer’s instructions. Real-time PCR were carried out in Step One Plus Real-Time PCR Systems (Applied Biosystems) by using Fast start SYBR Green Master according to the manufacturer’s instructions. The threshold cycle of each reaction was determined and normalized to that of GAPDH internal control.

The following primers were used in the RT-PCR reactions:

*GAPDH_F*: CTGCACCACCAACTGCTTAG,

*GAPDH_R*: AGGTCCACCACTGACACGTT,

*NOS2_F*: CCTGAGCTCTTCGAAATCC,

*NOS2_R*: AGGATGTTGTAGCGCTGGAC,

*NOX1_F:* CACAAGAAAAATCCTTGGGTCA,

*NOX1_R:* GACAGCAGATTGCGACACACA,

*NOX2_F*: GCCCAAAGGTGTCCAAGCT,

*NOX2_R*: TCCCCAACGATGCGGATAT,

*NOX4_F:* CCTCAACTGCAGCCTTATCC,

*NOX4_R:* CAACAATCTCCTGGTTCTCC,

*GSTP1_F*: ATGACTATGTGAAGGCACTG,

*GSTP1_R*:AGGTTCACGTACTCAGGGGA,

*SOD1_F*: CCTCTATCCAGAAAACACGG,

*SOD1_R*: CCAAACGACTTCCAGCG,

*NRF2_F*:TACTCCCAGGTTGCCCACA,

*NRF2_R*: CATCTACAAACGGGAATGTCTGC,

*GR_F*: AACATCCCAACTGTGGTCTTCAGC,

*GR_R*: TTGGTAACTGCGTGATACATCGGG,

*GPX3_F*: CGGGGACAAGAGAAGTCG,

*GPX3_R*: CCCAGAATGACCAGACCG,

*ALDH2_F*: CTACACACGCCATGAACCTG,

*ALDH2_R*: CAACCACGTTTCCAGTTG

### Immunofluorescence and analysis of ROS production

2 × 10^5^ cells were plated on a slide in 12-well plate in DMEM supplemented with 10% FBS, allowed them to grow for 24 h, washed with PBS and fixed with 4% PFA for 10–30 min at room temperature. The PFA was removed, cells washed with PBS, and blocked in a buffer containing 2% BSA, 0.1% Triton X-100 in PBS. After 5 min incubation at 4 °C, cells were rinsed with PBS, and incubated with an appropriate primary antibody during 1 hour at 37 °C. Cells were washed with PBS once, and incubated with a secondary antibody during 1 h at room temperature in the dark and washed with PBS. For the analysis of ROS production by DHE staining cells were treated as above, and after PFA was removed, cells were washed with PBS, and 1 ml of 20 μM DHE was added to on a slide. Cells were incubated at the room temperature with rotation during 5 min in the dark. DHE was removed, cells rinsed once with PBS.

Coverslips were mounted on a glass slides after Fluoroshield Mounting Medium with DAPI was added to the cells. For DHE detection images were observed at wavelengths range between 520 and 610 nm. For DAPI, GFP and Alexia Flour detection, images were observed in DAPI, 488 and 532 channels respectively. Images were acquired on a Confocal Leica SPE DM4000B microscope with 63x oil-immersion objective. Image intensities were adjusted and quantifications were carried out with LAS X software.

To test the effect of anti-oxidants on the level of ROS production, the culture medium was supplemented either with 100 μM tempol, or with 2 nM SKQ1, or with 20 μM Phox-I2, or with 500 nM GKT137831 3 h before the transfection; the ROS production analysis was carried out 24 h post-transfection.

### Cell cycle analysis

3 × 10^6^ cells were collected and washed in PBS. The pellet was resuspended in 1 ml of PBS and 3 ml of ice cold 95% ethanol was added slowly. The mixture was incubated for 30 min at 4 °C. 12 ml PBS was added till final volume of 15 ml and spun at 1.500 g for 10 min. Pellet was washed with 15 ml of PBS. Cells were counted, distributed in aliquots containing 1 million cells and spun at 1.500 g for 10 min. A pellet was resuspended in 1 ml DAPI stain solution (1 μg/ml DAPI, 0.1% Triton x100) and incubated on ice for 30 min. LSRFortessa cell analyzer (BD Biosciences) was used in FACS experiments for the cell cycle analysis, with excitation and emission wavelength of 359 nm and 461 nm respectively.

### Analysis of cell viability, apoptosis and mitochondrial dysfunction

Cell viability was measured using the XTT kit in accordance with the manufacturer’s instructions. A number of viable cells were estimated with methylene blue staining read on a Victor 1420 multilabel counter. Annexin V Apoptosis Detection Kit eFluor® 450 was used to assay cell apoptosis following the manufacturer’s instructions and analyzed by flow cytometry at LSRFortessa. The Mitochondrial ToxGlo™ Assay kit (Promega) was used for analysis of the mitochondrial dysfunction by measuring cellular ATP level according to the manufacturer’s instructions.

### Data availability

All the data generated or analysed during this study are included in this article and its Supplementary Information Files.

## Electronic supplementary material

Supplementary Information

Table S1

Table S2

Table S3

Table S4
