# Supplementary material for: Tumor suppressor NPRL2 induces ROS production and DNA damage response
Source: Sci Rep. 2017 Nov 10;7:15311. doi: 10.1038/s41598-017-15497-0 (PMC5681675; doi:10.1038/s41598-017-15497-0)
Supplement: Supplementary file 1 — Supplementary Information [file 41598_2017_15497_MOESM1_ESM.pdf]

SUPPLEMENTARY INFORMATION

Tumor suppressor NPRL2 induces ROS production and DNA damage response

Yinxing Ma[1], Licia Silveri[1], John LaCava[2,3] and Svetlana Dokudovskaya[1, *]

1- CNRS UMR 8126, Université Paris-Sud 11, Institut Gustave Roussy, 114, rue Edouard Vaillant, 94805, Villejuif, France

2- Laboratory of Cellular and Structural Biology, The Rockefeller University, New York, New York, USA.

3- Institute for Systems Genetics and Department of Biochemistry and Molecular Pharmacology, New York University School of Medicine, New York, New York 10016, USA

* Correspondence to: Svetlana Dokudovskaya;

E-mail: svetlana.dokudovskaya@igr.fr

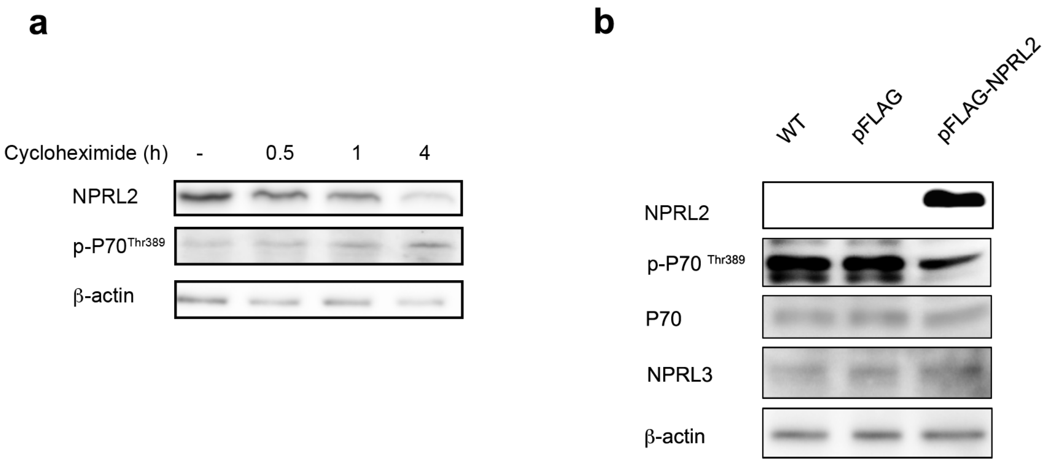

Figure S1. Effects of the NPRL2 overexpression on mTORC1 activity.

(**a**) Cycloheximide treatment induces mTORC1 activity via downregulation of NPRL2 expression level. HEK293 cell lines stably expressing FLAG-NPRL2-GFP were treated with 0.5 mM cycloheximide for indicated times, whole cell extracts were prepared and analyzed by Western blot with indicated antibodies.

(**b**) NPRL2 overexpression inhibits mTORC1 activity, but does not change NPRL3 protein level. Whole cell extracts were prepared from wild type HEK 293 cells (WT) and cells transformed with 1 μg pFLAG or pFLAG-NPRL2 plasmids for 24 h. WCE were analyzed by Western blot with indicated antibodies.

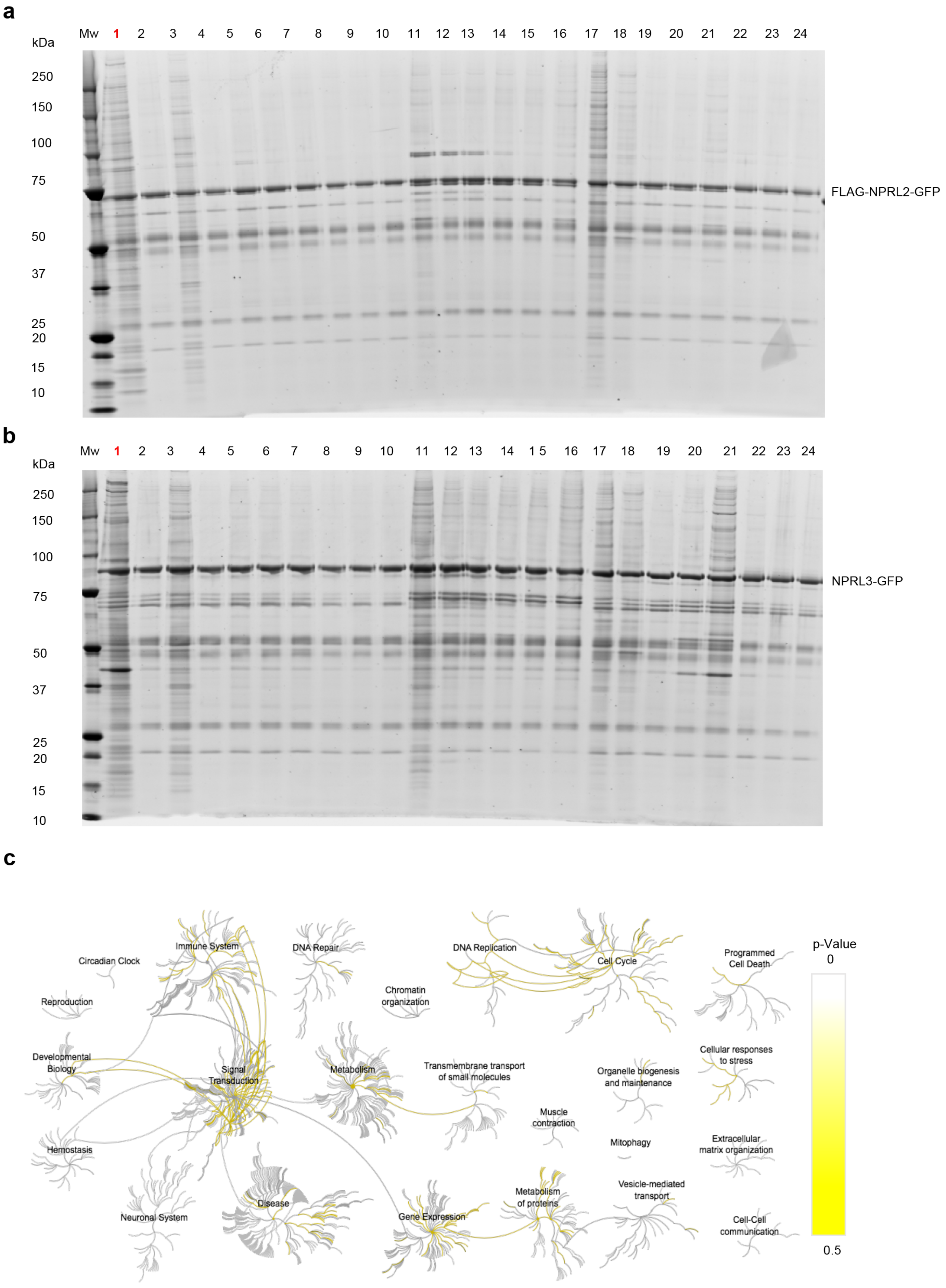

Figure S2. GATOR1 proteome.

(**a, b**) HEK293 cells stably expressing FLAG-NPRL2-GFP (a) or NPRL3-GFP (b) were immunoprecipitated using 24 different extraction buffers as described in Methods. Co-precipitating proteins were resolved by SDS-PAGE and visualized by Coomassie blue staining. A condition used for large scale proteome analysis is indicated in red.

(**c**) Distribution of co-purified proteins according to the overrepresentation in cellular pathways. Proteins appeared in NPRL2 proteome were analyzed by Reactome pathway database (www.reactome.org).

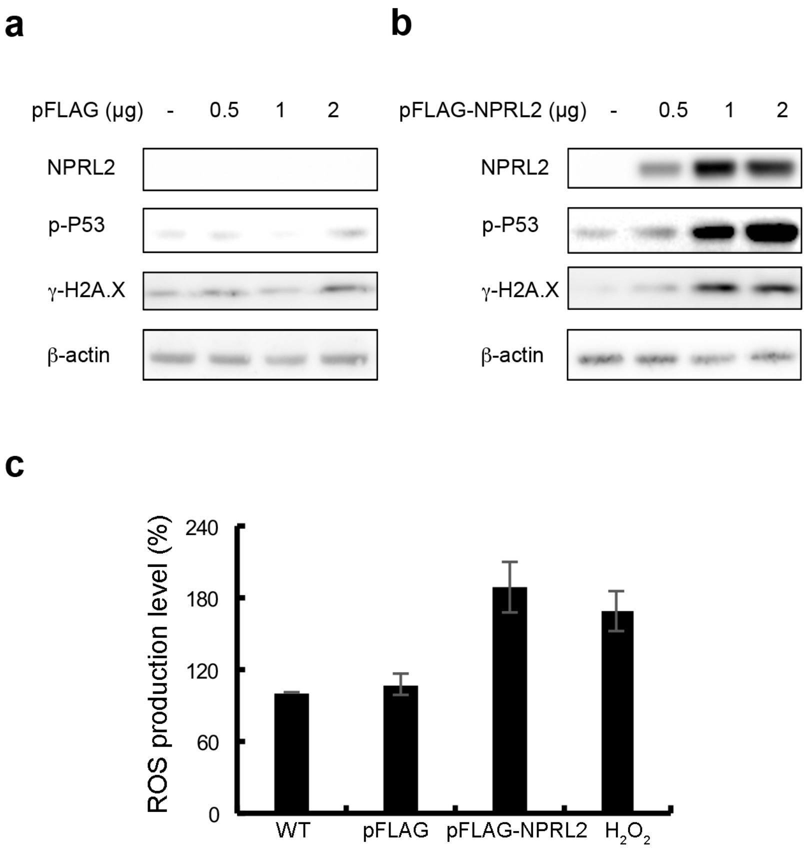

Figure S3. NPRL2 overexpressionin induces p53 phosphorylation and γ-H2A.X level in a dosage dependent manner, and ROS production comparable to the level achieved during H_2O_2 treatment. HEK293 cells were transfected for 24 h with empty vector (pFLAG) (**a**) or pFLAG-NPRL2 (**b**) at indicated concentrations. The whole cell extracts were analyzed by Western blot with indicated antibodies**.** **(c)** ROS production was estimated by measuring DHE level in non-treated HEK293 cells (NT), and in cell transformed with 1µg pFLAG or pFLAG-NPRL2 plasmids for 24 h or cells treated with 50 µM H_2O_2 for 1h.
